# Fully biological production of adipic acid analogs from branched catechols

**DOI:** 10.1038/s41598-020-70158-z

**Published:** 2020-08-07

**Authors:** Nicholas S. Kruyer, Natalia Wauldron, Andreas S. Bommarius, Pamela Peralta-Yahya

**Affiliations:** 1 School of Chemical and Biomolecular Engineering, Georgia Institute of Technology, Atlanta, GA 30332 USA; 2 School of Chemistry and Biochemistry, Georgia Institute of Technology, Atlanta, GA 30332 USA

**Keywords:** Enzymes, Synthetic biology, Metabolic engineering

## Abstract

Microbial production of adipic acid from lignin-derived monomers, such as catechol, is a greener alternative to the petrochemical-based process. Here, we produced adipic acid from catechol using catechol 1,2-dioxygenase (CatA) and a muconic acid reductase (MAR) in *Escherichia coli*. As the reaction progressed, the pH of the media dropped from 7 to 4-5 and the muconic acid isomerized from the *cis,cis* (ccMA) to the *cis,trans* (ctMA) isomer. Feeding experiments suggested that cells preferentially uptook ctMA and that MAR efficiently reduced all muconic isomers to adipic acid. Intrigued by the substrate promiscuity of MAR, we probed its utility to produce branched chiral diacids. Using branched catechols likely found in pretreated lignin, we found that while MAR fully reduced 2-methyl-muconic acid to 2-methyl-adipic acid, MAR reduced only one double bond in 3-substituted muconic acids. In the future, MAR’s substrate promiscuity could be leveraged to produce chiral-branched adipic acid analogs to generate branched, nylon-like polymers with reduced crystallinity.

## Introduction

Adipic acid is used in the production of nylon 6,6, a polyamide present in carpets, textiles and molded plastics. In 2016, the global production of adipic acid was ~ 3.3 million tons per year, with all adipic acid being produced from petroleum^1^. This process generated nearly 10% of global nitrogen oxide emissions^2^.

Renewable production of adipic acid provides a greener alternative, and can be initiated from a variety of feedstocks, including simple sugars and lignin-derived aromatics^1^. Independent of the feedstock used, today, the renewable production of adipic acid is a semi-biological process, combining bioproduction of muconic acid followed by its chemical hydrogenation to adipic acid^3,4^. For example, *Pseudomonas putida* has been engineered to convert pretreated lignin to *cis,cis*-muconic acid (ccMA) at 100% yield from detectable monomers^5^. *Escherichia coli* has a maximum theoretical yield of 83% from glucose through the shikimate pathway^1^, but experimentally a top yield of 22% has been achieved^6^. Purification of ccMA from the fermentation broth requires four unit operations to achieve the required purity (99.8%) at 81.4% yield^7^. Reduction of ccMA to adipic acid is performed using platinum, rhodium or palladium catalysts, with catalyst cost around $0.30 per kg of adipic acid^7–9^, ~ 19% of the current market value of adipic acid from petroleum^10^. Direct production of adipic acid from glucose through a reverse adipate degradation pathway has been achieved, but has a lower maximum theoretical yield (67%)^11^.

The fully biological production of adipic acid would eliminate (1) the need for a chemical reactor, (2) the catalyst cost, and (3) the cost of purifying muconic acid prior to chemical hydrogenation. Indeed, a recent techno-economic analysis (TEA) accounting for both fixed and variable costs concluded that a fully biological route to adipic acid from glucose would result in an adipic acid price point of $1.36/kg, while fully chemical and hybrid biological and chemical routes would result in price points of $1.56/kg and $1.48/kg^10^. A separate TEA showed that switching feedstock from glucose to lignin monomers reduced adipic acid minimum selling price by 50% due to increased productivity and decreased feedstock cost^12^. Taken together, lignin is a more desirable feedstock than sugars for adipic acid production.

Lignin depolymerisation results in a number of aromatic compounds, including catechol, a key intermediate in the production of adipic acid from both glucose and lignin-derived aromatics such as ferulic acid and p-coumaric acid^1^. Niu et al*.* achieved the fully biological production of adipic acid from lignin-derived aromatics in *P. putida*. Specifically, 4-hydroxybenzoic acid was converted to catechol and subsequently 3-ketoadipoyl-CoA via the β-ketodipate pathway. Using three heterologous enzymes and one endogenous thioesterase, 3-ketoadipoyl-CoA was converted to adipic acid with 17.4% molar yield^13^. Sun et al. developed a shorter pathway in *E. coli*, where glucose is metabolized to catechol via the shikimate pathway and catechol is converted to adipic acid using catechol-1,2-dioxygenase (CatA) and an enoate reductase previously shown to reduce muconic acid to adipic acid (i.e. muconic acid reductase, MAR)^14^. This pathway achieved 80.6 µg/L/hr of adipic acid^15^. Therefore, a better understanding of the CatA-MAR enzyme cascade can be directly applied to adipic acid production pathways that route through catechol.

**Figure 1:**
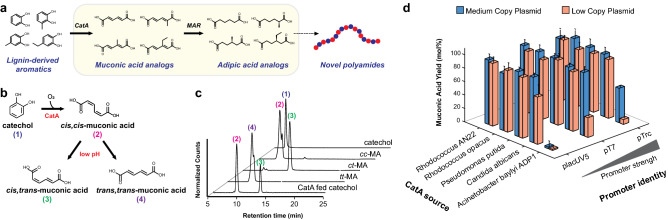
Muconic acid production from catechol. (**a**) Schematic for the bioproduction of adipic acid analogs. CatA: catechol-1,2-dioxygenase, MAR: muconic acid reductase. (**b**) Catechol to muconic acid conversion using CatA. At low pH, *cis,cis*-muconic acid (cc-muconic acid) can isomerize to *cis,trans*-muconic acid (ct-muconic acid) and *trans,trans*-muconic acid (tt-muconic acid). (**c**) Liquid chromatography/mass spectrometry (LC/MS) chromatogram of *E. coli* expressing *Rhodococcus* sp. AN22 CatA fed 1 g/L of catechol after 23 h incubation resulted in ccMA and ctMA. No ttMA was observed. Also shown, standards for catechol (6.8 min), ccMA (9.4 min), ctMA (13.6 min) and ttMA (9.6 min). Sample data for LC/UV absorbance spectra found in Supplementary Fig. S4. (**d**) Optimization of muconic acid production by screening CatAs from five different sources using three different promoter strengths and in low and medium copy plasmids. Experiments were run in triplicate and error bars represent the standard deviation from the mean.

Here, we optimized the CatA-MAR enzyme cascade in *E. coli* and discovered that the cascade is capable of converting branched catechols to adipic acid analogs (Fig. 1a). First, we optimized muconic acid production from catechol by screening and optimizing the expression of CatAs from five different organisms. Next, with the optimal CatA in hand, we maximized adipic acid production by optimizing the co-expression of two known MARs. Interestingly, during muconic acid production, the media acidified leading to the isomerization of ccMA to *cis*,*trans*-muconic acid (ctMA). To determine the extent to which MAR reduced ctMA, we fed ccMA and ctMA to cells expressing MAR and observed preference to reduce ctMA over ccMA. However, when we fed ccMA and ctMA to the cell lysate, MAR showed no preference between ccMA and ctMA. This data suggests that ctMA is preferentially transported into the cell over ccMA. Given the promiscuity of MAR, we explored its utility by feeding the CatA-MAR cascade branched catechols likely found in lignin^16^ to produce chiral-branched diacids. We found that MAR reduced one double bond in muconic acid analogs with short alkyl chains at the 3-position and both double bonds of 2-methyl-muconic acid to produce 2-methyl-adipic acid. The promiscuity of MAR highlights the potential utility of the CatA-MAR enzyme cascade in lignin valorization, as lignin depolymerisation results in a heterogeneous mixture of aromatics.

## Materials and methods

### Reagents

All compounds were purchased from Sigma-Aldrich unless noted. Catechol (135011), ccMA (15992, ≥ 97% purity), *trans,trans*-muconic acid (ttMA) (M90003, 98% purity), 3-methyl-catechol (Alfa Aesar, A12324), 4-methyl-catechol (TCI, M0413) and 4-ethyl-catechol (Alfa Aesar, A12048). To synthesize ctMA, 5 mM ccMA was acidified to pH 4–4.5 and incubated for 1 h at 70 °C to achieve complete isomerization^17^. Solutions of ccMA were adjusted to pH 7 to avoid isomerization. All muconic acid solutions were prepared fresh prior to use.

### Strains and plasmids

Gene sequences for the five CatA homologs (*Pseudomonas putida*—WP_010954549, *Acinetobacter baylyi* ADP1—CAG68305, *Candida albicans*—KGQ97177, *Rhodococcus sp.* AN22—BAH56722, and *Rhodococcus opacus*—3HGI_A) and the two MAR homologs (*Bacillus coagulans*—AEO99944 and *Clostridium acetobutylicum*—AEI32805) were codon optimized for *E. coli* and commercially synthesized. High translation rate ribosome binding sites (RBSs) were designed using the RBS Calculator^18,19^ and added to the synthesized genes via PCR (Supplementary Fig. S1). The genes carrying the high translation RBS were cloned into pBbA1a, pBbA1c, pBbS1a, pBbB1c, pBbA5c, pBbS5c, pBbB5a, pBbA7a and pBbS7a^20^ at BglII/ BamHI via Gibson assembly to generate CatA and MAR expression plasmids. To clone CatA and MAR as an operon, RBS2-CatA was amplified from pBbS1a_CatA-AN22 (pNK45), RBS1-MAR-BC was amplified from pNK102, and RBS1-MAR-CA was amplified from pBbA1a_MAR-CA (pNK146). The PCR products were cloned into pBbA1a at BglII/BamHI via Gibson assembly to generate pBbA1a_MAR-BC_CatA-AN22 (pNK111) and pBbA1a_MAR-CA_CatA-AN22 (pNW1). Clones were confirmed via sequencing using primers NK18 (pTrc), NK19 (placUV5), NK20 (pT7) and NK22.

### Screening CatA for muconic acid production from catechol

Overnight cultures of *E. coli* DH10B transformed with pTrc and placUV5 CatA expression plasmids (pNK53-72) or *E. coli* BL21 (DE3) transformed with pT7 CatA expression plasmids (pNK73-82) were diluted to OD_600_ = 0.1 in 5 mL of M9 minimal media (Difco 248,510: 33.9 g/L Na_2_HPO_4_, 15.0 g/L KH_2_PO_4_, 2.5 g/L NaCl, 5.0 g/L NH_4_Cl, 2 mM MgSO_4_, 100 µM CaCl_2_, supplemented with 0.5% glucose) and 100 mg/L ampicillin or 50 mg/L chloramphenicol. Cultures were incubated (37 °C, 250 RPM) until reaching OD_600_ = 0.4–0.6. CatA expression was induced using 500 µM IPTG and 1 g/L (9.1 mM) catechol was fed to the cultures. Cultures were incubated for 23 h at 37 °C, 250 RPM. After incubation, cultures were centrifuged and the supernatant analysed via liquid chromatography, mass spectrometry (LC–MS).

### Screening MAR for adipic acid production from muconic acid

Overnight cultures of *E. coli ΔiscR* transformed with MAR expression plasmids (pNK102, pNK146-149, pNK160-163, pNW5, pNW8 and pNW10) were diluted to OD_600_ = 0.1 in 5 mL of M9 minimal media with 100 mg/L ampicillin or 50 mg/L chloramphenicol and supplemented with 1 mg/L thiamine. Cultures were incubated (37 °C, 250 RPM) until reaching OD_600_ = 0.4–0.6. MAR expression was induced using 500 µM IPTG and fed either 500 µM ccMA, ctMA or ttMA. Cultures were then transferred to anaerobic conditions (anaerobic tubes with argon headspace and sealed using chlorobutyl rubber stoppers and Parafilm) and incubated for 24 h at 30 °C, 250 RPM. After incubation, cultures were centrifuged and the supernatant analysed via LC–MS.

### Cell lysate MAR activity on muconic acid isomers

Overnight cultures of *E. coli ΔiscR* transformed with MAR expression plasmids (pNK102, pNK146) or *E. coli ΔiscR* (control) were diluted to OD_600_ = 0.1 in 5 mL of LB media (Difco 240210: 10 g/L tryptone, 5 g/L yeast extract, 5 g/L NaCl ) supplemented 100 mg/L ampicillin when needed. Cultures were incubated (37 °C, 250 RPM) until reaching OD_600_ = 0.4–0.6. MAR expression was induced with 500 µM IPTG and cultures were transferred to anaerobic conditions and incubated for 24 h at 30 °C, 250 RPM. After incubation, 500 µL of 10 × lysis solution (10 mg/mL lysozyme, 0.5% Tween-20, 10 mM DTT, 10 U/mL DNase, 25 mM MgCl_2_) was added to the culture using a needle through the rubber stopper to maintain low oxygen conditions within the tube. Cells were lysed for 1 h at 30°, 250 RPM. After lysis, 500 µL of 7 mM ccMA, ctMA or ttMA (final concentration 583 µM) was added using a needle through the rubber stopper. The cultures were incubated for 24 h (30 °C, 250 RPM), centrifuged, and the supernatant analysed via LC–MS. The control corroborated that neither media nor lysis solution reduced muconic acid to adipic acid.

### Production of adipic acid analogs from lignin-derived aromatic monomers

Overnight cultures of *E. coli ΔiscR* transformed with CatA-MAR enzyme cascades (pNK111 or pNW1) were diluted to OD_600_ = 0.1 in 5 mL of M9 minimal media with 100 mg/L ampicillin or 50 mg/L chloramphenicol and supplemented with 1 mg/L thiamine and incubated at 37 °C, 250 RPM until reaching OD_600_ = 0.4–0.6. Co-expression of CatA and MAR was induced using 500 µM IPTG and fed either 1 mM catechol or substituted catechol (3-methyl-catechol, 4-methyl-catechol or 4-ethyl-catechol). Cultures were incubated for 2 h at 30 °C, 250 RPM before transferring to anaerobic conditions and cultured for 22 h. After incubation, the cultures were centrifuged and the supernatant was analyzed via LC/MS.

### Chemical analysis

**Figure 2:**
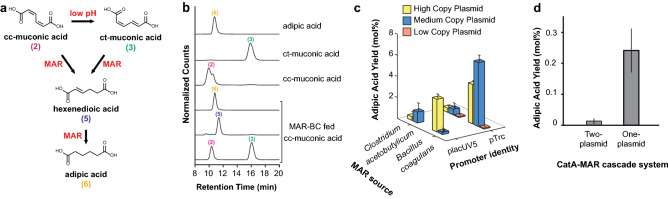
Adipic acid production from muconic acid and catechol. (**a**) Reduction of *cis,cis*-muconic acid (cc-muconic acid) and *cis,trans*-muconic acid (ct-muconic acid) to adipic acid by muconic acid reductase (MAR). (**b**) LC/MS chromatograms of *E. coli* expressing *Bacillus coagulans* MAR fed 500 µM ccMA after 24 h incubation results in adipic acid, hexenedioic acid as well as unreacted *cis,cis*-muconic acid and *cis,trans*-muconic acid. Also shown, standards for adipic acid (*m*/*z* 146; 10.8 min), *cis,trans*-muconic acid (*m*/*z* 142; 16.1 min) and *cis,cis*-muconic acid (*m*/*z* 142; 10.0 min). (**c**) Adipic acid yields (mol%) obtained by screening MARs from two different sources using two different promoter strengths and in low, medium and high copy plasmids. (**d**) Adipic acid yields obtained from feeding 500 mg/L catechol to *E. coli* co-expressing *Rhodococcus* sp. AN22 CatA and *Bacillus coagulans* MAR in a two and one plasmid system. For **c** and **d**, experiments were run in triplicate and error bars represent the standard deviation from the mean.

LC/MS analysis was completed using an Agilent 1100/1260 series system equipped with a 1260 ALS autosampler, a multi-wavelength detector (MWD) and a 6120 Single Quadrupole LC/MS with a Poroshell 120 SB-C18 3.0 mm × 50 mm × 2.7 μm column and an electrospray ion source. Column temperature was kept constant at 28 °C. LC method was based on method developed by Sun et al*.*^21^*.* LC conditions for muconic acid analysis: Solvent A—water with 0.1% formic acid, Solvent B—methanol. Gradient: 1 min 95%:5%:0.2 (%A:%B:flow rate in ml/min), 15 min ramp to 75%:25%:0.2, 1 min ramp to 95%:5%:0.2, 18 min 95%:5%:0.2. LC conditions for analogs of muconic acid and adipic acid were: Solvent A—water with 0.1% formic acid, Solvent B—acetonitrile. Gradient: 1 min 95%:5%:0.1, 25 min ramp to 75%:25%:0.1, 1 min ramp to 95%:5%:0.1, 18 min 95%:5%:0.1. *For CatA screening* (Fig. 1d), catechol and muconic acid were quantified using a multi-wavelength detector (MWD) (274 nm catechol, 260 nm muconic acid). Quantification was done using the area under the peak for the corresponding MWD signal. *For MAR screening* (Fig. 2d), MS acquisition was done in negative ion mode for all samples and using selected ion monitoring (SIM). Catechol (*m*/*z* 109.1), ccMA (*m*/*z* 141.1), ctMA (*m*/*z* 141.1), ttMA (*m*/*z* 141.1), hexenedioic acid (*m*/*z* 143.1), adipic acid (*m*/*z* 145.1), 3-methyl-catechol (*m*/*z* 123.1), 2-methyl-muconic acid (*m*/*z* 155.1), 2-methyl-hexenedioic acid (*m*/*z* 157.1), 2-methyl-adipic acid (*m*/*z* 159.1), 4-methyl-catechol (*m*/*z* 123.1), 3-methyl-muconic acid (*m*/*z* 155.1), 3-methyl-hexenedioic acid (*m*/*z* 157.1), 3-methyl-adipic acid (*m*/*z* 159.1), 4-ethyl-catechol (*m*/*z* 137.1), 3-ethyl-muconic acid (*m*/*z* 169.1), 3-ethyl-hexenedioic acid (*m*/*z* 171.1) and 3-ethyl-adipic acid (*m*/*z* 173.1). Quantification was done using the area under the peak for the corresponding SIM signal. Areas were converted to concentrations for catechol, ccMA, ctMA, ttMA and adipic acid, using standard curves generated using commercial standards. There are no commercial standards for 2-methyl-muconic acid, 3-methyl-muconic acid, 3-ethyl-muconic acid, hexenedioic acid, 2-methyl-hexenedioic acid, 3-methyl-hexenedioic acid, 3-ethyl-hexenedioic acid and 2-methyl-adipic acid, MS counts are reported.

## Results and discussion

### Catechol to muconic acid conversion

CatAs from *Pseudomonas putida, Pseudomonas aeruginosa, Acinetobacter calcoaceticus, Acinetobacter baylyi,* and *Acinetobacter baylyi* ADP1 have been used to produce muconic acid in *E. coli*^6,21–23^. We compared the *E. coli* performance of CatAs from *P. putida, Acinetobacter baylyi* ADP1 (both dimers with moderate specific activity—*P. putida* 22.4 µM/min/mg), *Candida albicans* (dimer with a higher specific activity, 63 µM/min/mg), *Rhodococcus* sp. AN22 (a monomer) and *Rhodococcus opacus* (structurally well studied)^8,24–28^ (Supplementary Figs. S2, S3). The CatAs were expressed from three promoters with different strengths in low and medium plasmid copy number (Fig. 1b–d, Fig. S4). All CatAs performed similarly independent of promoter strength and plasmid copy number, reaching almost 100% catechol to muconic acid conversion after 24 h (Fig. 1d). Of note, *Acinetobacter baylyi* ADP1 CatA showed < 10% conversion in half of the tested conditions despite similar protein expression (Supplementary Fig. S5). Active site alignment of the ADP1 CatA and *P. putida* CatA revealed that at position 76 ADP1 CatA codes for a proline, while *P. putida* CatA codes for an alanine (Supplementary Fig. S6). A proline to alanine mutation at this position has been shown to increase ADP1 CatA specific activity 10 fold^27^. Therefore, P76 in ADP1 CatA may lead to the underperformance observed when compared to the other CatA homologs. For all subsequent experiments, we used *Rhodococcus* sp. AN22 CatA as it performed well and it is a monomer, reducing the metabolic load of the system.

*E. coli* expressing AN22 CatA converts catechol to ccMA as the major product. However, we also detected the thermodynamically stable isomer ctMA, which is likely produced from ccMA after media acidification after 24 h growth, which reaches a pH 4.3 after starting at pH ~ 7 (Fig. 1b,c). We measured the maximum ccMA to ctMA isomerization rate to be between pH 3–5 (Supplementary Fig. S7), with previous literature confirming the maximum at pH 4^17^. No isomerization of ctMA to ttMA was observed, which is consistent with the literature^17^.

### Muconic acid to adipic acid conversion

We compared the *E. coli* performance of *Bacillus coagulans* MAR (MAR-BC) and *Clostridium acetobutylicum* MAR (MAR-CA)^14^ expressed from two promoters with different strengths in low, medium and high plasmid copy number (Fig. 2a–c). MAR-BC achieved a 6.1% conversion of 500 µM ccMA to adipic acid using the pTrc promoter from a medium-copy plasmid after a 24 h anaerobic growth. This is lower than Sun et al*.* who used batch fermentation to convert 2.8 mM of ccMA to adipic acid (18.0% total conversion) using MAR-CA^15^. We rationalize our lower percent conversion in comparison by the use of minimal M9 media supplemented only with 0.5% glucose rather than a modified M9 medium, which contains 0.5% yeast extract, 0.25% glucose and 1% glycerol that aids in higher protein expression and increased cell density.

### Adipic acid production from catechol

The experimental conditions for the CatA-MAR enzyme cascade required balancing the molecular oxygen requirement of the CatA reaction with the oxygen sensitivity of MAR. Thus, fermentations were run as a two-stage batch process with a 2 h aerobic stage followed by a 22-h anaerobic stage^29^. The CatA-MAR cascade was tested in a two- and one-plasmid system. As Fig. 2d shows, the one-plasmid system resulted in 1.6 mg/L of adipic acid after 24 h, or a 0.241% molar yield from the fed 1 g/L catechol, an 18-fold improvement over the two-plasmid set up.

### MAR muconic acid isomer preference

**Figure 3:**
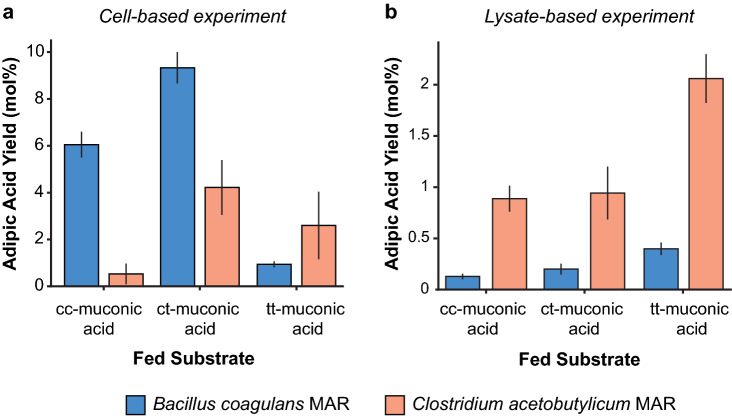
Muconic acid reductase substrate preference (**a**) Adipic acid production of *Escherichia coli* expressing *Bacillus coagulans* muconic acid reductase (MAR) or *Clostridium acetobutylicum* MAR when feeding 500 µM of *cis,cis*-muconic acid (cc-muconic acid), *cis,trans*-muconic acid (ct-muconic acid), *trans,trans*-muconic acid (tt-muconic acid) isomers. (**b**) Adipic acid production of lysed *E. coli* expressing *B. coagulans* MAR and *C. acetobutylicum* MAR fed 583 µM of muconic acid isomers. All experiments were performed in triplicate and error bars represent the standard deviation from the mean.

At pH < 7, ccMA isomerized to ctMA, and both isomers were present at a roughly equal molar ratio in the fermentation broth (Fig. 2b). Hypothesizing that MAR may not reduce ctMA as efficiently as ccMA, we fed ccMA, ctMA and, for completion, ttMA to cells expressing MAR and to a cell lysate expressing MAR. In the cell-based experiment, both MARs showed a preference to reduce ctMA over ccMA with MAR-CA showing an eightfold preference. MAR-BC also showed a higher adipic acid yield than MAR-CA; 11.5-fold in the case of ccMA, and twofold in the case of ctMA (Fig. 3a). In the cell lysate-based experiment, both MARs reduced ccMA and ctMA to the same extent. Interestingly, MAR-CA showed a higher adipic acid yield than MAR-BC (Fig. 3b). MAR-CA also showed a preference for ttMA, which is consistent with previous literature^14^. Of note, the overall yields in the cell lysate experiment were lower than the cell-based experiment, likely due to enzyme deactivation from cell lysis solution components and the reduced co-factor concentration. Taken together, we rationalize the in vivo MAR substrate preference for ctMA to be the result of increased membrane permeability for ctMA over ccMA. We rationalize the higher in vivo activity of MAR-BC over MAR-CA to the fact that once a muconic acid isoform enters the cell the MAR experiences a higher localized substrate concentration. Previously, it has been suggested that MAR-BC has a lower substrate affinity but higher catalytic activity than MAR-CA^14^. Such MAR-BC enzymatic characteristics would fit the observed results.

### MAR muconic acid analog preference

**Figure 4:**
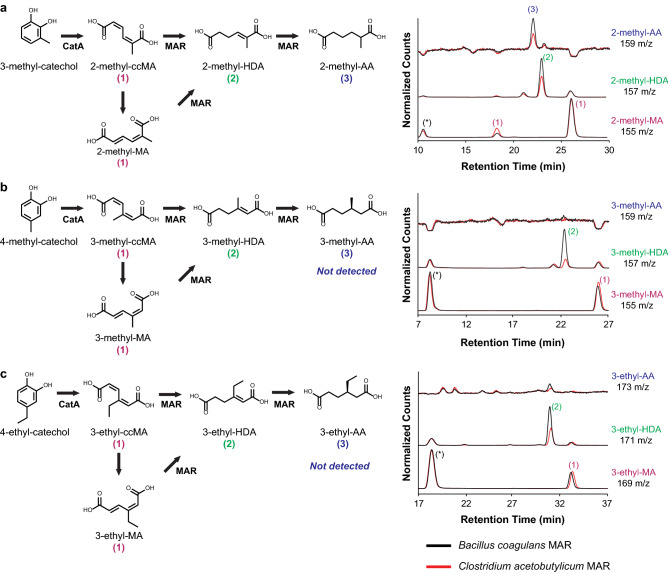
Production of muconic acid and adipic acid analogs. Reaction schematic and LC/MS chromatograms of *Escherichia coli* co-expressing *Rhodococcus* sp. AN22 CatA and either *B. coagulans* MAR or *C. acetobutylicum* MAR and fed 1 mM (**a**) 3-methyl-catechol results in 2-methyl hexenedioic acid and 2-methyl-adipic acid; (**b**) 4-methyl-catechol results in 3-methy hexenedioic acid, and (**c**) 4-ethyl-catechol results in 3-ethyl hexenedioic acid. *: Peaks visible in MS but not in UV channel, thus not muconic acid analog peaks (Supplementary Fig. S8).

Given that MAR reduces all muconic acid isomers, we investigated the extent to which the CatA-MAR cascade can produce branched adipic acid analogs from alkyl substituted catechols likely found in pretreated lignin^16^. We confined our analysis to commercially available catechols previously shown to be oxidized by CatA: 3-methyl-catechol (3MC), 4-methyl-catechol (4MC), and 4-ethyl-catechol (4EC)^30^. CatA oxidized 3MC, 4MC and 4EC to 2-methyl-muconic acid, 3-methyl-muconic acid and 3-ethyl-muconic acid, respectively (Fig. 4a–c). MARs singly reduced 3-methyl-muconic acid and 3-ethyl-muconic acid to 3-methyl-hexenedioic acid and 3-ethyl-hexenedioic acid. Only 2-methyl-muconic was doubly reduced to produce 2-methyl-adipic acid by MAR (Fig. 4a). While the substrate specificities of MAR-BC and MAR-CA were similar, MAR-BC led to higher yields of the singly and fully reduced muconic acid analogs (Fig. 4).

CatA oxidation of 3MC resulted in two 2-methyl-muconic acid peaks. Presumably, *cis,cis*-2-methyl-muconic acid isomerized to the *cis,trans* isomer over the course of the reaction (Fig. 4a, Supplementary Fig. S8). Interestingly, CatA oxidation of 4MC or 4EC resulted in a single peak for 3-methyl-muconic acid and 3-ethyl-muconic acid, respectively (Fig. 4b,c, Supplementary Fig. S8). Given the steric crowding between the ketone group and the alkyl chains at position 3 in muconic acid in the *cis,cis* configuration, we presume that only *cis,trans*-3-methyl-muconic acid and *cis–trans*-3-ethyl-muconic acid are present in the media.

## Conclusions

The fully biological production of adipic acid from catechol, a pretreated lignin monomer, was achieved by screening CatA from different sources and optimizing its co-expression with MAR in *E. coli* to produce adipic acid at 1.6 mg/L or a 0.241% molar yield. The muconic acid yields presented in this work were lower than in previous studies, which we attribute to the use of batch fermentation rather than a biocatalysis set up and the use of minimal media rather than rich media, which makes it problematic to calculate yields.

A closer study of oxygen sensitivity differences between MAR-BC and MAR-CA may help to engineer a more oxygen tolerant enzyme that will be useful in the production of adipic acid from lignin-derived monomers as oxygen is both a substrate (CatA) and an inhibitor (MAR) of the process. Engineering MAR for oxygen tolerance will help improve catechol to adipic acid yields in the future.

A key finding of this work is the use of MAR to produce branched adipic acid analogs. Thus, the CatA-MAR cascade could be used to convert lignin-derived monomers to chiral branched dicarboxylic acids that may give tuneable properties to nylon-6,6 like polymers. Application of the enzyme cascade to a variety of lignin-derived monomers demonstrates increased utility as a lignin valorization approach.

## Supplementary information

Supplementary Information.

## Abbreviations

CatA: Catechol-1,2-dioxygenase
MAR: Muconic acid reductase
ccMA: *cis,cis-*Muconic acid
ctMA: *cis,trans*-Muconic acid
ttMA: *trans,trans*-Muconic acid

## References

[1] Kruyer NS, Peralta-Yahya P (2017). Metabolic engineering strategies to bio-adipic acid production. Curr. Opin. Biotechnol..

[2] Polen T, Spelberg M, Bott M (2013). Toward biotechnological production of adipic acid and precursors from biorenewables. J. Biotechnol..

[3] Kohlstedt M (2018). From lignin to nylon: cascaded chemical and biochemical conversion using metabolically engineered *Pseudomonas putida*. Metab. Eng..

[4] van Duuren JBJH (2020). Limited life cycle and cost assessment for the bioconversion of lignin-derived aromatics into adipic acid. Biotechnol. Bioeng..

[5] Salvachúa D (2018). Bioprocess development for muconic acid production from aromatic compounds and lignin. Green. Chem..

[6] Niu W, Draths KM, Frost JW (2002). Benzene-free synthesis of adipic acid. Biotechnol. Progr..

[7] Vardon DR (2016). cis,cis-Muconic acid: separation and catalysis to bio-adipic acid from nylon-6,6 polymerization. Green. Chem..

[8] Vardon DR (2015). Adipic acid production from lignin. Energy. Environ. Sci..

[9] Claypool JT, Raman DR (2013). Development and validation of a technoeconomic analysis tool for early-stage evaluation of bio-based chemical production processes. Bioresour. Technol..

[10] Gunukula S, Anex RP (2017). Techno-economic analysis of multiple bio-based routes to adipic acid. Biofuel. Bioprod. Biorefining.

[11] Zhao M (2018). Metabolic engineering of *Escherichia coli* for producing adipic acid through the reverse adipate-degradation pathway. Metab. Eng..

[12] Johnson CW (2019). Innovative chemicals and materials from bacterial aromatic catabolic pathways. Joule.

[13] Niu W (2020). Direct biosynthesis of adipic acid from lignin-derived aromatics using engineered *Pseudomonas putida* KT2440. Metab. Eng..

[14] Joo JC (2017). Alkene hydrogenation activity of enoate reductases for an environmentally benign biosynthesis of adipic acid. Chem. Sci..

[15] Sun J, Raza M, Saun X, Yuan Q (2018). Biosynthesis of adipic acid via microaerobic hydrogenation of *cis,cis*-muconic acid by oxygen sensitive enoate reductase. J. Biotechnol..

[16] Schutyser W (2018). Chemicals from lignin: an interplay of lignocellulose fractionation, depolymerisation, and upgrading. Chem. Soc. Rev..

[17] Carraher JM, Pfennig T, Rao RG, Shanks BH, Tessonnier J-P (2017). cis, cis-Muconic acid isomerization and catalytic conversion to biobased cyclic-C6-1,4-diacid monomers. Green. Chem..

[18] Espah Borujeni A, Salis HM (2016). Translation initiation is controlled by RNA folding kinetics via a ribosome drafting mechanism. J. Am. Chem. Soc..

[19] Salis HM, Mirsky EA, Voigt CA (2009). Automated design of synthetic ribosome binding sites to control protein expression. Nat. Biotechnol..

[20] Lee TS (2011). BglBrick vectors and datasheets: a synthetic biology platform for gene expression. J. Biol. Eng..

[21] Sun XX (2013). A novel muconic acid biosynthesis approach by shunting tryptophan biosynthesis via anthranilate. Appl. Environ. Microbiol..

[22] Lin Y, Sun X, Yuan Q, Yan Y (2014). Extending shikimate pathway for the production of muconic acid and its precursor salicylic acid in *Escherichia coli*. Metab. Eng..

[23] Wu W (2017). Lignin valorization: two hybrid biochemical routes for the conversion of polymeric lignin into value-added chemicals. Sci. Rep..

[24] Matsumura E, Ooi S, Murakami S, Takenaka S, Aoki K (2004). Constitutive synthesis, purification and characterization of catechol 1,2-dioxygenase from the aniline-assimilating bacterium *Rhodococcus* sp. AN-22. J. Biosci. Bioeng..

[25] Tsai S-C, Li Y-K (2007). Purification and characterization of a catechol 1,2-dioxygenase from a phenol degrading *Candida albicans* TL3. Arch. Microbiol..

[26] Matera I (2010). Catechol 1,2-dioxygenase from the gram-positive *Rhodococcus opacus* 1CP: quantitative structure/activity relationship and the crystal structures of nativee and catechols adducts. J. Struct. Biol..

[27] Han L (2015). Engineering catechol 1,2-dioxygenase by design for improving the performance of the cis, cis-muconic acid and synthetic pathway in *Escherichia coli*. Sci. Rep..

[28] Sanakis Y, Mamma D, Christakopoulos P, Stamatis H (2003). Catechol 1,2-dioxygenase from *Pseudomonas putida* in organic media: an electron paramagnetic resonance study. Int. J. Biol. Macromol..

[29] Raj K (2018). Biocatalytic production of adipic acid from glucose using engineered *Saccharomyces cerevisiae*. Metab. Eng. Commun..

[30] Cha C-J (2006). Catechol 1,2-dioxygenase from *Rhodococcus rhodochrous* N75 capable of metabolizing alkyl-substituted catechols. J. Microbiol. Biotechnol..

