# Supplementary material for: Fully biological production of adipic acid analogs from branched catechols
Source: Sci Rep. 2020 Aug 7;10:13367. doi: 10.1038/s41598-020-70158-z (PMC7414886; doi:10.1038/s41598-020-70158-z)
Supplement: Supplementary file 1 — Supplementary Information. [file 41598_2020_70158_MOESM1_ESM.pdf]

Fully Biological Production of Adipic Acid Analogs from Branched Catechols

Nicholas S. Kruyer[1], Natalia Wauldron[1], Andreas S. Bommarius[1,2], and Pamela Peralta-Yahya[1,2]

Supplementary Information

Table of Contents

Supplemental Methods	Page
Strains and Plasmids	SI1
SDS-PAGE analysis of CatA expression	SI1
Western blot analysis of CatA expression	SI1
**Table S1**. Table of strains	SI2
**Table S2.** Table of plasmids	SI2
**Table S3.** Table of primers	SI4
**Figure S1**. Calculated translation initiation rates with synthetic RBS	SI5
**Figure S2**. Rationale for CatA choice	SI5
**Figure S3**. CatAs sequence identity	SI6
**Figure S4**. Sample Liquid chromatography/ultraviolet (LC/UV) spectra for conversion of catechol to muconic acid by CatA-AN22	SI6
**Figure S5**. Expression of CatA homologs	SI7
**Figure S6**. Active site alignment of CatA from *P. putida* and *A. baylyi* ADP1	SI7
**Figure S7**. Isomerization of *cis,cis*-MA to *cis,trans*-muconic acid as a function of pH	SI8
**Figure S8**. Comparison of mass spectrometry and UV active peaks for Figure 5	SI9
**Sequences**	SI10
**References**	SI13

Supplemental Methods

Strains and Plasmids. N-terminal 6x His Tags were added to CatA genes (pNK58-60 and pNK62) via PCR. The vector pBbS1a was amplified using primers NK153 and NK154. The His-tagged CatAs were cloned into pBbS1a backbone using Gibson Assembly to generate pNK167-171. Clones were confirmed via sequencing using primers NK18 and NK22.

SDS-PAGE analysis of CatA expression. Overnight cultures of *E. coli* DH10B transformed with His-tagged CatA expression plasmids (pNK167-171) were diluted to OD_{600} = 0.1 in 5 mL of LB media with 100 mg/L ampicillin. Cultures were incubated (37°C, 250 RPM) until reaching OD_{600} = 0.4-0.6. CatA expression was induced using 500 μM IPTG and incubated for 4 hours (37°C, 250 RPM). After incubation, cultures were centrifuged and the pellets resuspended in 1x phosphate buffered saline (PBS: Teknova P0195 – 135 mM NaCl, 2.7 mM KCl, 4.3 mM Na_2HPO_4, 1.4 mM NaH_2PO_4, pH 7) to a cell density of 40 mg/mL. 1 mL of cell suspension was lysed using sonication. The sonication was performed on ice, and the sonication protocol was 8 on-off cycles of 30 seconds. After sonication, the lysed cells were centrifuged. The total protein concentration of the soluble fraction was measured by the absorbance at 280nm using a NanoDrop Lite (Thermo). Protein concentrations were standardized to 2 mg/mL total protein by diluting in PBS to a total volume of 50 μL. After addition of 10 μL of 6x SDS loading dye, the samples were heated at 95°C for 15 minutes. 20 μL of each sample was loaded to both 12% Bis-Tris SDS-PAGE gels (Invitrogen) along with the BenchMark™ His-tagged Protein Standard (Invitrogen LC5606). Two identical gels were run in parallel in MES SDS running buffer (Invitrogen) at 200 V for 45 minutes at 4°C. One gel was stained with Coomassie Blue and one was used for Western Blot.

Western blot analysis of CatA expression. Proteins in the SDS-PAGE gel were transferred to nitrocellulose paper using ThermoFisher iBlot™ Transfer Stack. The primary antibody was anti-polyhistidine antibody produced in mouse (Sigma H1029) and the secondary antibody was anti-mouse Fc produced in goat (Sigma A3638). After antibody incubation, the nitrocellulose paper was stained with BCIP/NBT (Sigma BC5655).

Table S1. Table of strains

Strain #	Description	Source
PPY252	*Escherichia coli* DH10B	Invitrogen
PPY2108	*Escherichia coli* BL21 (DE3)	NEB
PPY1535	*Escherichia coli* K-12 BW25113 Δ*iscR*	Baba et al [4]

Table S2. Table of plasmids

Strain Number	Plasmid Name	Description	Source
PPY2109	pBbA1a	p15a (medium copy), pTrc, ampR	Lee et al [3]
PPY2110	pBbA1c	p15a, pTrc, CmR	Lee et al [3]
PPY2111	pBbS1a	SC101 (low copy), pTrc, ampR	Lee et al [3]
PPY2112	pBbB1c	BBR1 (high copy), pTrc, CmR	Lee et al [3]
PPY2113	pBbA5c	p15a, lacUV5, CmR	Lee et al [3]
PPY2114	pBbS5c	SC101, lacUV5, CmR	Lee et al [3]
PPY2115	pBbB5a	BBR1, lacUV5, ampR	Lee et al [3]
PPY2116	pBbA7a	p15a, T7, ampR	Lee et al [3]
PPY2117	pBbS7a	SC101, T7, ampR	Lee et al [3]
PPY1521	pNK45	pBbS1a-CatA-AN22*	This Study
PPY1626	pNK53	pBbA1a-CatA-putida	This Study
PPY1627	pNK54	pBbA1a-CatA-albicans	This Study
PPY1628	pNK55	pBbA1a-CatA-opacus	This Study
PPY1629	pNK56	pBbA1a-CatA-AN22	This Study
PPY1630	pNK57	pBbA1a-CatA-ADP1	This Study
PPY1631	pNK58	pBbS1a-CatA-putida	This Study
PPY1632	pNK59	pBbS1a-CatA-albicans	This Study
PPY1633	pNK60	pBbS1a-CatA-opacus	This Study
PPY1634	pNK61	pBbS1a-CatA-AN22	This Study
PPY1635	pNK62	pBbS1a-CatA-ADP1	This Study
PPY1636	pNK63	pBbA5c-CatA-putida	This Study
PPY1637	pNK64	pBbA5c-CatA-albicans	This Study
PPY1638	pNK65	pBbA5c-CatA-opacus	This Study
PPY1639	pNK66	pBbA5c-CatA-AN22	This Study
PPY1640	pNK67	pBbA5c-CatA-ADP1	This Study
PPY1641	pNK68	pBbS5c-CatA-putida	This Study
PPY1642	pNK69	pBbS5c-CatA-albicans	This Study
PPY1643	pNK70	pBbS5c-CatA-opacus	This Study

PPY1644	pNK71	pBbS5c-CatA-AN22	This Study
PPY1645	pNK72	pBbS5c-CatA-ADP1	This Study
PPY1646	pNK73	pBbA7a-CatA-putida	This Study
PPY1647	pNK74	pBbA7a-CatA-albicans	This Study
PPY1648	pNK75	pBbA7a-CatA-opacus	This Study
PPY1649	pNK76	pBbA7a-CatA-AN22	This Study
PPY1650	pNK77	pBbA7a-CatA-ADP1	This Study
PPY1651	pNK78	pBbS7a-CatA-putida	This Study
PPY1652	pNK79	pBbS7a-CatA-albicans	This Study
PPY1653	pNK80	pBbS7a-CatA-opacus	This Study
PPY1654	pNK81	pBbS7a-CatA-AN22	This Study
PPY1655	pNK82	pBbS7a-CatA-ADP1	This Study
PPY1744	pNK102	pBbA1a-MAR-BC	This Study
PPY1776	pNK111	pBbA1a-MAR-BC_CatA-AN22**	This Study
PPY1967	pNW1	pBbA1a-MAR-CA_CatA-AN22**	This Study
PPY1973	pNW5	pBbB1c-MAR-BC	This Study
PPY1976	pNW8	pBbB5a-MAR-BC	This Study
PPY1983	pNK146	pBbA1a-MAR-CA	This Study
PPY1984	pNK147	pBbB5a-MAR-CA	This Study
PPY1985	pNK148	pBbB1c-MAR-CA	This Study
PPY1986	pNK149	pBbA5c-MAR-CA	This Study
PPY1987	pNW10	pBbA5c-MAR-BC	This Study
PPY2087	pNK159	pBbA1c-MAR-BC	This Study
PPY2088	pNK160	pBbS5c-MAR-BC	This Study
PPY2089	pNK161	pBbS5c-MAR-CA	This Study
PPY2090	pNK162	pBbS1a-MAR-BC	This Study
PPY2091	pNK163	pBbS1a-MAR-CA	This Study
PPY2104	pNK167	pBbS1a-6His-CatA-putida	This Study
PPY2105	pNK168	pBbS1a-6His-CatA-albicans	This Study
PPY2106	pNK169	pBbS1a-6His-CatA-opacus	This Study
PPY2107	pNK170	pBbS1a-6His-CatA-ADP1	This Study
PPY2122	pNK171	pBbS1a-6His-CatA-AN22	This Study

All plasmids have RBS1 unless indicated with *
* = single gene that had RBS2
** = operon plasmid, MAR had RBS1 and CatA had RBS2.

Table S3. Table of primers

Primer name	Sequence
NK18	gacaattaatcatccggctcg
NK19	CACTTTATGCTTCCGGCTCG
NK20	cgcgaaattaatacgactcactatag
NK22	ccgacaaacaacagataaaacg
NK26	GGATCCTTAACCTTCTTGCAGTGCGC
NK27	GGATCCTTATAACTTAATCTCCGCATCCTGACG
NK28	GGATCCTTATGCCGGATCTAAAACAAAATTATAGG
NK29	GGATCCTTATGCTTCCGGGTCCAGC
NK30	GGATCCTTACACTGCCAGGCGCG
NK31	AGATCTATGCACCATCACCATCACCATACTGTTAAAATCAGCCATACAGC
NK32	AGATCTATGCACCATCACCATCACCATTCACAGGCGTTTACAGAAAGCG
NK33	AGATCTATGCACCATCACCATCACCATACCACAACAGAAAGTCCTACAGC
NK35	AGATCTATGCACCATCACCATCACCATGAGGTTAAGATTTTTAATACCCAAGATG
NK40	AGATCCAGACGATAGCGACCTACCCAGGAGGAAATAACATGACTACAATGGAGAATCC GACC
NK41	GAACCCAACATTTATTATAATAAGGAGTTAAACAATGACTACAATGGAGAATCCGACC
NK43	CGGATAACAATTTCAGAATTCAAAAGATCTAGATCCAGACGATAGCGACC
NK47	GCCTGGAGATCCTTACTCGAGTTTGGGGATCCTTATGCTTCCGGG
NK44	CGGATAACAATTTCAGAATTCAAAAGATCTGAACCCAACATTTATTATAATAAGG
NK63	AGATCCAGACGATAGCGACCTACCCAGGAGGAAATAACATGCACCATCACCATCACC
NK64	AGATCCAGACGATAGCGACCTACCCAGGAGGAAATAACATGACTGTTAAAATCAGCCAT ACAGC
NK65	GCCTGGAGATCCTTACTCGAGTTTGGATCCTTAACCTTCTTGCAGTGC
NK66	AGATCCAGACGATAGCGACCTACCCAGGAGGAAATAACATGTCACAGGCGTTTACAGAA AGC
NK67	GCCTGGAGATCCTTACTCGAGTTTGGATCCTTATAACTTAATCTCCGC
NK68	AGATCCAGACGATAGCGACCTACCCAGGAGGAAATAACATGACCACAACAGAAAGTCC
NK69	GCCTGGAGATCCTTACTCGAGTTTGGATCCTTATGCCGGATCTAAAACAAAATTATAGG
NK70	AGATCCAGACGATAGCGACCTACCCAGGAGGAAATAACATGGAGGTTAAGATTTTTAAT ACCC
NK71	GCCTGGAGATCCTTACTCGAGTTTGGATCCTTACACTGCCAGG
NK75	GCCTGGAGATCCTTACTCG
NK82	AGATCCAGACGATAGCGACCTACCCAGGAGGAAATAACATGAAATACAAAAAGCTATTT GAAACTGTG
NK83	AGATCCAGACGATAGCGACCTACCCAGGAGGAAATAACATGAACAAATACAAGAAATTA TTTGAACCAATC
NK86	GTGAGCGGATAACAATTTCAGAATTC
NK88	GGTCTGTTTCCTGTGTGAAATTGTTATCCTTACAGATTTGCCGCGACTTCG

NK89	AGGATAACAATTTCACACAGGAAACAGACCGAACCCAACATTTATTATAATAAGGAGTTAAACAATG
NK153	GGATCCAAACTCGAGTAAGGATC
NK154	CTTTTGAATTCTGAAATTGTTATCCGC

Name	Nucleotide Sequence	Predicted Translation Initiation Rate (a.u.) [1-2]
RBS1	agatccagacgatagcgacctacccaggaggaaataac	6161.40
RBS2	gaacccaacatttattataataaggagttaaaca	74751.05

Figure S1. Calculated translation initiation rates with synthetic RBS

Organism	Quaternary structure	Specific Activity (μmol/min/mg)	Logic for Testing
Acinetobacter baylyi ADP1	Dimer	Only Crude Reported (Han, 2015 [5]; Niu, 2002 [6])	Similar to used to achieve highest cis,cis-muconic acid titers in *Escherichia coli* to date (Niu, 2002 [6]). Mutational study of active site (Han, 2015 [5]).
Candida albicans	Dimer	63 (Tsai, 2007 [7])	CatA with the highest reported specific activity
Pseudomonas putida	Dimer	22.4 (Vardon, 2015 [8])	Natively expressed in *Pseudomonas putida*, an organism used for lignin monomer upgrading.
Rhodococcus opacus	Dimer	n.d. (Matera, 2010 [9])	Enables structural insight through crystal structures with a variety of substrates
Rhodococcus sp. AN22	Monomer	35 (Matsumura, 2004 [10])	CatA monomer with highest reported specific activity

Figure S2. Rationale for CatA choice

	P. putida	***R. opacus***	***R. sp AN22***	***C. albicans***	***A. baylyi ADP1***
P. putida		28%	27%	30%	56%
R. opacus			69%	26%	26%
R. sp AN22				27%	27%
C. albicans					26%
A. baylyi ADP1					

Figure S3. CatAs sequence identity

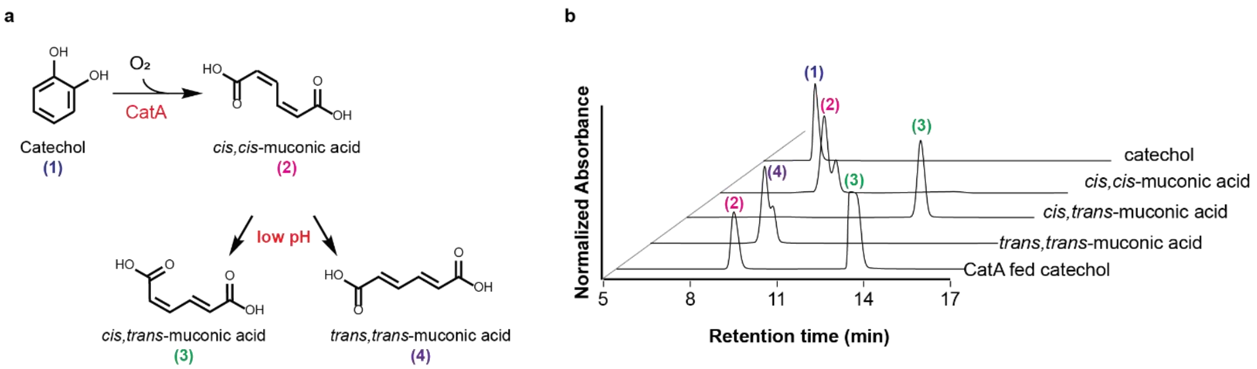

Figure S4: Liquid chromatography-UV (LC/UV) spectra for conversion of catechol to muconic acid by *Rhodococcus* sp. AN22 CatA. (**a**) Conversion of catechol to *cis,cis*-muconic acid (ccMA) via CatA and isomerization of ccMA to *cis,trans*-muconic (ctMA) acid at low pH. (**b**) LC/UV spectra for standards of catechol, ccMA, ctMA, and *trans,trans*-muconic acid, and the supernatant of *E. coli* expressing CatA-AN22 from the pTrc promoter in a medium copy plasmid (pNK56) when fed catechol. Corresponds to data in **Figure 1c**.

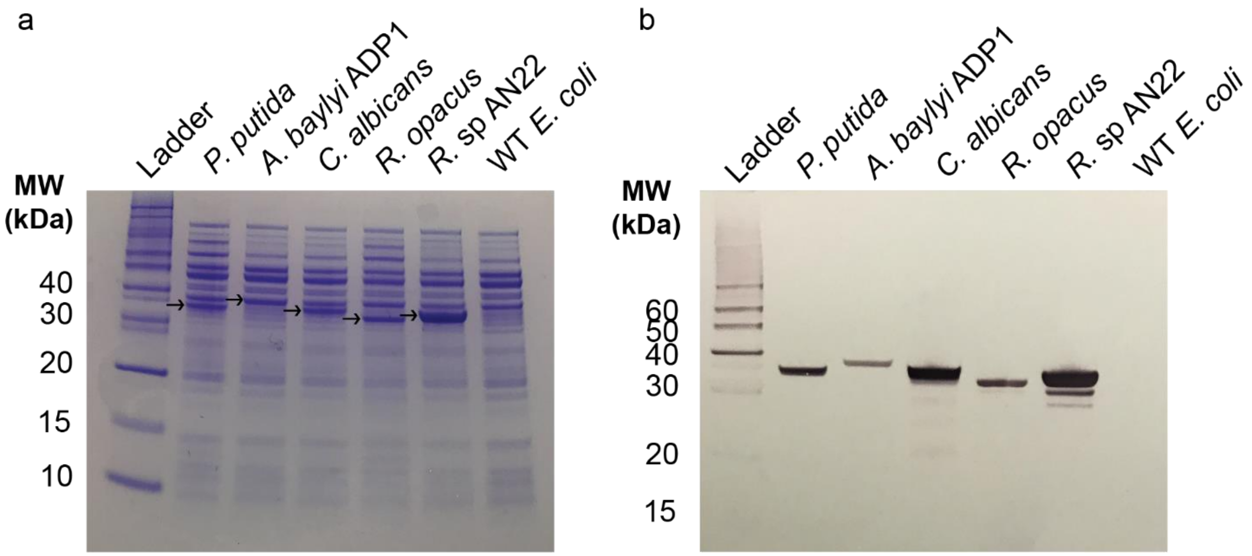

Figure S5: Expression of CatA homologs. (a) Coomassie stained SDS-PAGE gel for His$_6$-CatA homologs expressed under pTrc promoter, in low copy vector. **(b)** Western Blot His$_6$-CatA homologs. CatA Molecular Weight: *P. putida* – 34.3 kDa, *A. baylyi* ADP1 – 34.3 kDa, *C. albicans* – 33.8 kDa, *R. opacus* – 30.7 kDa, *R.* sp. AN22 – 31.6 kDa,

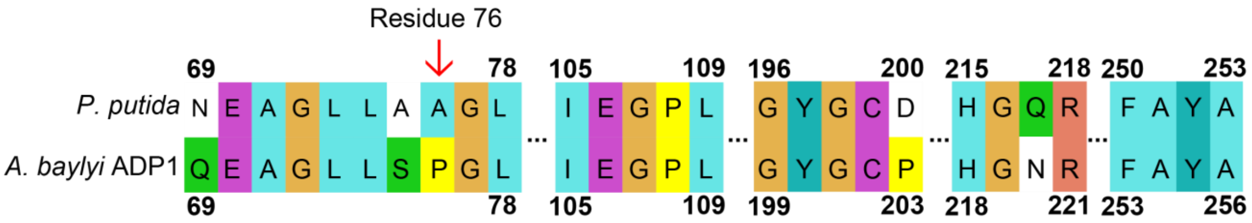

Figure S6: Active site alignment of *Pseudomonas putida* CatA and *Acinetobacter baylyi* ADP1 CatA. Active site residues from Han et al (2015) [5]. Colors represent different classes of amino acid. Key difference at residue 76 may account for conversion differences when using *P. putida* CatA and *A. baylyi* ADP1 CatA.

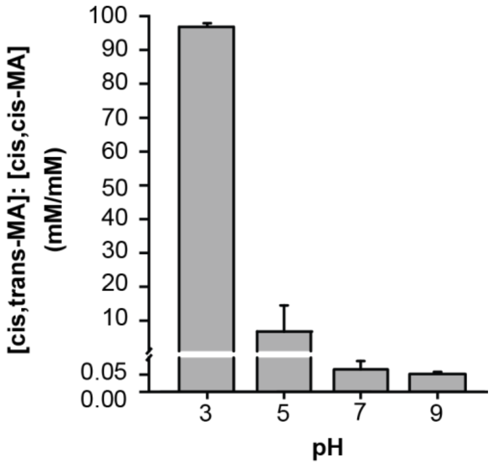

Figure S7: Isomerization of *cis,cis*-muconic acid to *cis,trans*-muconic acid as a function of pH. Ratio of concentration of ctMA to concentration of ccMA after 300 µM solution of ccMA was incubated for 24 hours at 37°C/250 RPM in M9 media of varying pH value.

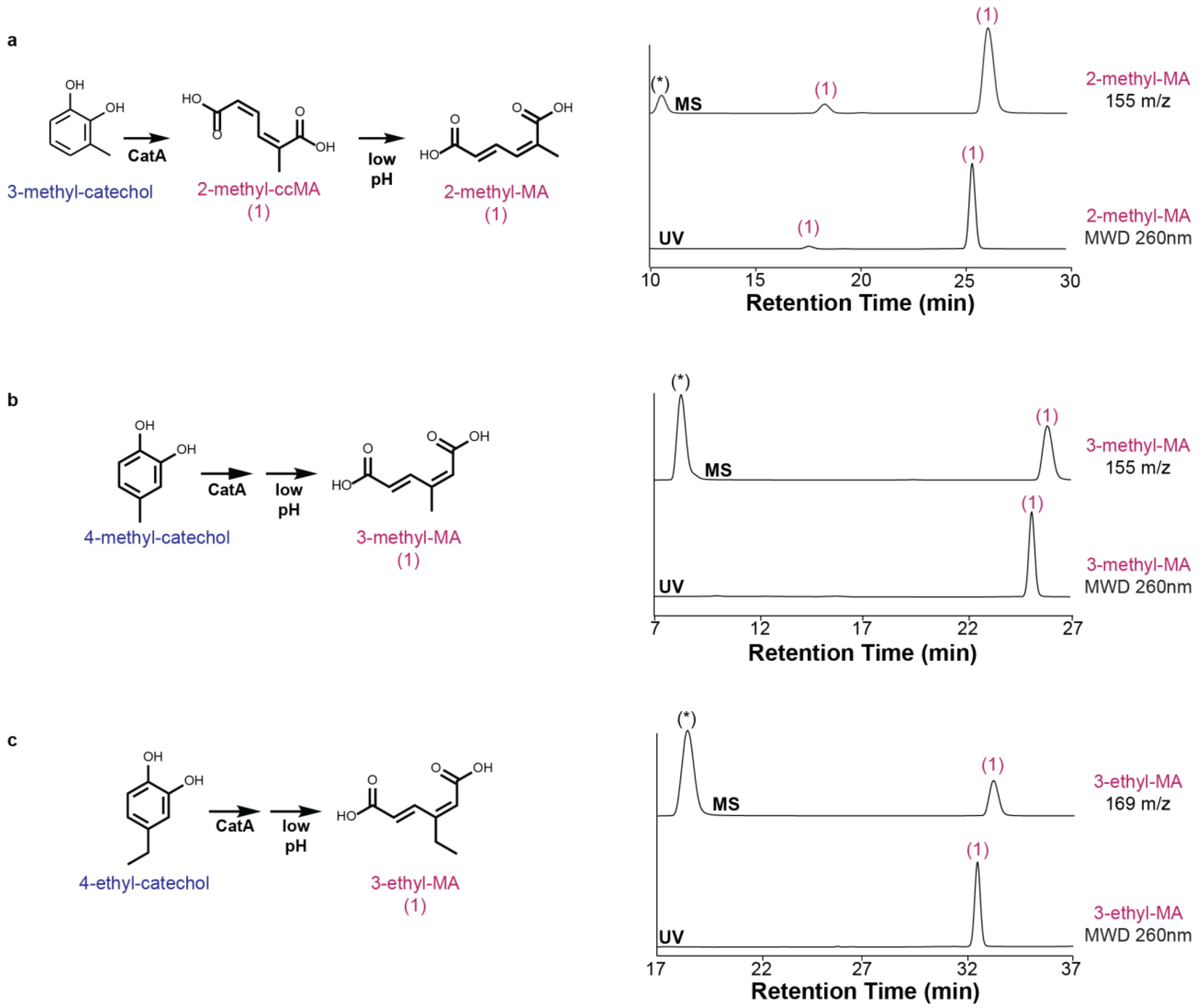

Figure S8: Comparison of MS and UV active peaks for Figure 5. (**a**) 2-methyl muconic acid (m/z 155) and UV trace. (**b**) 3-methyl-muconic acid (m/z 155) and UV trace. (**c**) 3-ethyl-muconic acid (m/z 169) and UV trace. All traces come from *E. coli ΔiscR* expressing CatA from *Rhodococcus* sp. AN22 and MAR from *Bacillus coagulans* fed 1 mM of substituted catechol after 2-hr aerobic and 22-hr anaerobic incubation. Peaks appearing in both SIM and UV channels are muconic acid analogs. *: Peaks only present in SIM (likely media components). Difference in peak times between UV trace and SIM due to volume (tubing) distance between MWD and MS detectors.

Sequences

> *Pseudomonas putida* CatA (WP_010954549)

ATGACTGTTAAAATCAGCCATACAGCAGATATTCAGGCATTCTTTAATAGAGTTGCAGG
TTTAGATCATGCAGAAGGCAATCCGCGTTTTAAACAGATTATTCTGCGCGTTTTACAAG
ATACAGCACGTTTAATTGAAGATTTAGAAATTACCGAAGATGAATTTTGGCATGCAGTG
GATTATCTGAACCGTCTTGGCGGACGCAACGAAGCCGGTTTACTGGCAGCAGGCCTTG
GTATTGAACATTTTCTGGATCTGTTACAGGACGCAAAAGACGCAGAAGCAGGTCTGGG
CGGTGGTACCCCGCGCACCATTGAAGGCCCGCTGTATGTTGCAGGTGCACCGCTGGC
ACAGGGCGAAGCACGCATGGATGATGGTACCGATCCGGGCGTTGTTATGTTTCTGCAA
GGTCAGGTATTTGACGCAGATGGTAAACCGCTGGCAGGCGCAACCGTTGATCTGTGGC
ATGCGAATACCCAGGGTACCTATAGCTATTTTGATAGCACCCAGAGCGAATTTAACCTG
CGCCGTCGTATTATTACCGATGCAGAAGGCCGTTATCGTGCACGCAGCATTGTGCCGA
GCGGCTATGGCTGTGATCCGCAGGGTCCGACCCAGGAATGTCTGGATCTGCTGGGCC
GTCATGGTCAGCGCCCGGCACATGTTCATTTCTTTATTAGCGCACCGGGTCATCGCCA
TCTGACCACCCAGATTAATTTTGCAGGTGATAAATATCTGTGGGATGATTTTGCGTATG
CAACCCGCGATGGTCTGATTGGTGAACTGCGCTTTGTGGAAGATGCGGCAGCAGCAC
GCGATCGCGGTGTGCAGGGTGAACGTTTTGCGGAACTGAGTTTTGACTTTCGCCTGCA
AGGCGCTAAAAGCCCGGATGCAGAAGCACGCAGCCATCGCCCGCGCGCACTGCAAGA
AGGTTAA

> *Acinetobacter baylyi* ADP1 CatA (CAG68305)

ATGGAGGTTAAGATTTTTAATACCCAAGATGTGCAAGACTTCCTGCGCGTTGCAAGCGG
CCTGGAACAAGAAGGCGGTAACCCGCGTGTGAAACAAATTATTCATCGCGTTCTGAGT
GATTTATATAAAGCAATTGAAGACTTAAACATTACCAGTGATGAATATTGGGCAGGTGT
GGCATATCTGAACCAGCTGGGCGCAAACCAGGAAGCAGGCCTGTTAAGCCCTGGCCT
GGGCTTTGATCATTATTTAGATATGCGCATGGATGCAGAAGATGCAGCACTGGGCATTG
AAAACGCAACACCGCGCACCATTGAAGGCCCGTTATATGTGGCAGGCGCACCTGAAAG
TGTGGGTTACGCACGCATGGATGATGGCAGCGATCCGAATGGTCATACCCTGATCCTG
CACGGCACCATTTTTGATGCAGATGGCAAACCTCTGCCGAACGCAAAAGTTGAAATCTG
GCACGCAAACACCAAGGGCTTCTATAGCCATTTTGATCCTACCGGCGAACAACAGGCA
TTTAACATGCGTCGCAGCATTATTACAGATGAGAACGGTCAGTATCGCGTTCGCACCAT
TCTGCCGGCAGGCTATGGCTGCCCGCCGGAAGGCCCGACCCAACAGCTGTTAAACCA
GTTAGGTCGCCACGGTAATCGCCCGGCACATATTCATTATTTTGTGAGCGCAGATGGC
CATCGCAAACTGACCACCCAGATTAACGTTGCAGGCGATCCGTATACCTATGATGATTT
TGCATACGCAACCCGCGAGGGCTTAGTTGTGGATGCAGTGGAACATACCGATCCGGAA
GCAATTAAAGCAAACGATGTGGAAGGCCCGTTTGCAGAAATGGTGTTCGATCTGAAACT
GACACGCCTGGTGGATGGTGTGGATAACCAGGTGGTTGATCGCCCGCGCCTGGCAGT
GTAA

> *Candida albicans* CatA (KGQ97177)

ATGTCACAGGCGTTTACAGAAAGCGTGAAAACAAGCCTGGGTCCGAACGCGACCCCTC
GCGCGAAGAAGCTGATTGCAAGTTTAGTGCAGCATGTGCATGACTTTGCACGCGAAAA
CCATCTGACAACAGAAGATTGGCTTTGGGGTGTGGATTTTATTAATCGCATTGGTCAAA
TGAGCGATAGTCGCCGCAATGAAGGCATTTTAGTGTGCGATATTATTGGTCTGGAAACC
CTGGTGGATGCCCTGACCAACGAAAGTGAACAAAGCAACCATACAAGTAGCGCGATTC
TGGGTCCGTTTTATTTACCGGACAGCCCGGTGTATCCGAATGGCGGTAGCATTGTTCA
GAAGGCAATTCCGACCGACGTTAAATGTTTTGTGCGCGGTAAAGTTACCGACACCGAA

GGTAAACCGTTAGGCGGTGCACAGCTGGAAGTTTGGCAGTGTAATAGCGCAGGTTTCT
ATAGCCAACAGGCAGATCATGATGGCCCGGAATTTAATCTGCGTGGTACATTTATTACC
GATGATGAAGGTAATTATAGCTTTGAATGCCTGCGGCCTACCAGCTACCCGATTCCGTA
TGATGGCCCGGCAGGTGACCTGCTGAAAATTATGGATCGCCATCCGAATCGTCCTAGC
CACATTCATTGGCGCGTGTCGCATCCGGGTTATCATACCCTGATTACCCAGATTTATGA
TGCGGAATGTCCGTATACCAACAACGATAGCGTGTATGCGGTTAAAGATGATATTATTG
TGCATTTTGAAAAGGTGGATAACGATGATAAAGACCTGGTGGGTAAAGTGGAATATAAA
CTGGATTATGATATTAGCCTGGCGACCGAAAGCAGTATTCAGGAAGCGCGCGCGGCAG
CGAAAGCGCGTCAGGATGCGGAGATTAAGTTATAA

> *Rhodococcus* sp. AN22 CatA (BAH56722)

ATGACTACAATGGAGAATCCGACCGCGCATGGTAGCGGCAATGCGGCAACCGATAAAT
TTAAAAGTGAACGCGTTACAAGCGACACAAGCGTTGAACGCGCGAGCGCAATTTATAA
AGATTTACTGGATGCGCTGGCAGGCATTGTTGATAAACATCAGGTGACCTATGATGAAT
ATAGAGTGTTAAAACAATGGCTGATTGATGTGGGAGAATATGGTGAATGGCCTTTATGG
TTAGATGTGTTTCTTGAACATGAGATTGAGAAAGTTCATTACAATCGCAAAGGTTTTACC
GGCACCAAAGGTAGTATTGAAGGCCCTTATTATGTGCCGGATAGCCCTAAATTACCGA
GTAAATGCACCATGCCTATGCGCGAGAAGGATAAAGTGGCGCCGCCTCTGGTGTTTAA
AGGTCAGGTGACCGATTTAGAAGGCAATGGTCTGCCGGGCGCAACCGTGGAACTGTG
GCATGCGGATGAAGAGGGCTTTTACAGCCAGTTTGCGCCGGGCATTCCTGAGTGGAAC
CTGCGCGGCACAGTGGAAGTGGATGAAAACGGCAACTTTGAAATTACAACACTGAAAC
CGGCGCCGTATCAGATTCCGAGCGATGGCCCGACCGGCTGGTTTATCAAAAGTTATGG
CGGCCACCCGTGGAGACCGGCGCATCTGCATCTTATGGTGAAAGCACCTGGCAAACG
CACCATTACCACCCAACTTTATTTTCAAGGCGGCGAATGGGTCGAAGATGATGTGGCAA
CAGCGGTTAAACCGGAACTGATTCTTGACCCGCAACCGAACGCGGATGGCGTGGCGG
AAGTGACCTATACCTTCGTGCTGGACCCGGAAGCATAA

> *Rhodococcus opacus* CatA (3HGI_A)

ATGACCACAACAGAAAGTCCTACAGCAGCAGGTTCAGGTAGCGCAGCAACAGATAAAT
TCAAAGCTGAACGCGCAACAGCAGATACAAGCCCGGAACGCCTGGCAGCAATTGCAAA
AGATGCATTAGGCGCACTGAACGATGTGATTTTAAAACATGGCGTTACCTATCCGGAAT
ATCGCGTGTTTAAACAATGGCTGATTGATGTGGGCGAAGGCGGTGAATGGCCATTATTT
CTGGATGTGTTTATTGAACATAGCGTGGAAGAAGTGCTGGCACGCAGTCGCAAAGGTA
CAATGGGTTCTATCGAAGGCCCGTATTATATTGAAAACAGCCCGGAACTGCCTAGCAAA
TGCACATTACCGATGCGCGAAGAAGATGAGAAAATTACCCCGCTGGTGTTTAGCGGCC
AGGTGACCGATCTGGATGGCAATGGCCTGGCAGGCGCGAAAGTGGAACTGTGGCATG
CAGATAACGATGGTTATTATAGCCAGTTTGCGCCTCATTTACCGGAATGGAACCTGCGC
GGCACCATCATTGCGGATGAAGAAGGTCGTTATGAAATTACCACCATTCAGCCGGCAC
CGTATCAGATTCCGACCGATGGCCCGACCGGCCAGTTTATTGAAGCGCAGAACGGTCA
TCCGTGGCGCCCGGCACATCTGCATCTGATTGTGAGCGCACCGGGCAAGGAAAGCGT
TACCACCCAGCTGTATTTTAAAGGCGGTGAATGGATTGATAGCGATGTGGCAAGCGCG
ACCAAACCGGAACTGATTCTGGACCCAAAGACCGGCGATGATGGCAAGAACTATGTGA
CCTATAATTTTGTTTTAGATCCGGCATAA

> ***Bacillus coagulans*** **MAR (AEO99944)**

ATGAAATACAAAAAGCTATTTGAAACTGTGAAAATAAGGAATGTGGAACTCAAAAATCGT
TATGCAATGGCACCAATGGGTCCGCTGGGTCTTGCCGATGCAGAAGGCGGTTTCAACC
AGCGCGGGATTGAGTATTATACAGCCCGTGCGCGCGGGGGAACCGCTCTGATTATTAC
CGGCGTCACTTTCGTTGATAATGAAGTGGAAGAGCACGGAATGCCAAACGTACCTTGC
CCGACCCATAACCCTGTCCATTTTGTCCGGACTTCCAAAGAAATGACAGAGCGCATCCA
TGCATATGATTCGAAAATTTTTCTGCAAATGAGCGCCGGTTTTGGCCGGGTGACGATCC
CGACAAACCTTGGCGAGTACCCGCCGGTTGCACCGTCGCCAATCCCGCATCGCTGGC
TGGATAAAACATGTCGCGAACTGACAGTTGAAGAAATTCATTCCATTGTCCGCAAATTC
GGGGATGGGGCGTTCAATGCGAAGCGCGCCGGATTTGACGGGGTGCAAATCCATGCT
GTGCACGAAGGCTATTTGCTCGACCAGTTTGCGATTGCGTTTTTCAACAAACGTACCGA
TGCATACGGTGGCCCGCTTGAAAATCGCCTTCGTTTTGCCCGGGAAATTGTCGAGGAA
ATTAAACAGCGCTGTGGCGAAGATTTTCCTGTGACGCTCCGCTTCAGCCCGAAAAGTTT
TATCAAGGATTGGCGGGAAGGGGCACTGCCTGGCGAGGAGTTTGAAGAAAAAGGCCG
CGATTTGGATGAAGGCATCGAGGCAGCAAAGCTGCTCGTTTCCTACGGCTATGATGCT
CTGGACGTCGATGTTGGTTCTTATGATTCATGGTGGTGGAGCCATCCTCCGATGTACCA
GAAGAAGGGGCTTTACATTCCGTATGCCAGGCTGGTGAAGGAAGCTGTCGATGTGCCT
GTCCTTTGCGCGGGCCGCATGGACAATCCGGATCTTGCACTTGCCGCACTGGAAGACG
GAGCATGTGATATTATCAGCTTGGGCCGCCCGTTATTGGCTGACCCGGATTACGTCAA
TAAGCTCCGAATCGGGCAGGTTGCCGATATCCGCCCGTGTCTGTCATGCCATGAAGGC
TGCATGGGTCGGATCCAGGAGTATTCTTCCTTAGGCTGCGCAGTGAATCCGGCTGCCT
GTCGAGAAAAAGAAGCAGCATTGACACCTGCTTTAAAAAAGAAACGCGTACTGATTGCA
GGCGGCGGCGTGGCCGGATGCGAAGCTGCCCGTGTGCTTGCATTGCGCGGCCATGAA
CCGGTCATTTTTGAAAAATCGAACCGTTTAGGCGGCAACTTGATCCCTGGCGGCGCAC
CTGATTTTAAAGAAGATGACCTGGCGCTTGTTGCCTGGTATGAGCATACGTTGGAACGC
CTTGGCGTAGAAATTCATTTGAATACTGCATTGACAAAAGAAGAAATTTTGGCTGCAAA
CGTGGATGCCGTGCTGATTGCAACGGGTTCGAATCCGAAAATTTTGCCGCTCGACGGA
AAAAACAAAGTATTTACAGCAGAAGATGTTTTGCTCGATAAAGTGGATGCCGGGCAACA
TGTTGTCATTGTCGGCGGCGGTCTTGTCGGCTGCGAACTGGCTTTGAACCTTGCAGAA
AAAGGAAAAGATGTCTCGCTTGTGGAAATGCAGGACAAACTGCTGGCAGTTAATGGTC
CGCTTTGCCACGCTAACTCGGACATGCTGGAAAGACTCGTACCGTTTAAAGGTGTTCAA
GTCTACACTTCTTCAAAAATAGTAGATACGACAGAAAAGACAGCCGTTGTGGATGTTGA
CGGCGAATTGCGTGAAATTGAAGCAGACAGCATTGTGCTCGCAGTCGGCTACTCGGCT
GAAAAATCACTCTATGAAGATTTAAAGTTTGAGGTTGCCGATCTTCATGTGGTTGGCGA
TGCCCGCAAGGTCGCAAACATCATGTATGCCATCTGGGATGCTTACGAAGTCGCGGCA
AATCTGTAA

> ***Clostridium acetobutylicum*** **MAR - AEI32805**

ATGAACAAATACAAGAAATTATTTGAACCAATCAAAATTGGAAAATGTGAAATCAAAAAC
CGTTTTGCATTAGCTCCAATGGGCCCTTTAGGACTAGCTGATAGTGAAGGTGGTTTCAA
CCAAAGAGGAATAGACTACTATACTGAAAGAGCAAAAGGTGGCACAGGATTAATAATAA
CAGGAGTTACCTTTGTAGATAATGAAGTTGAAGAACACGGAATGCCTAATTGTCCTTGT
CCAACACATAATCCAGTTCAATTCGTAAGAACTGGTAGAGAAATGACTGAAAGAATACA
CGCATACAATTCTAAAGTATTTTTACAAATGTCAGGTGGATTTGGTAGAGTTACTATACC
TACTAACTTAGGAGAATTTCCTCCAGTTGCCCCATCTCCAATTCAACATAGATGGCTTG
ACAAAACTTGTCGTGAACTTACAGTAGATGAAATTAAATCAATAGTTAAAAAATTTGGTG
AAGGAGCTTTTAATGCTAAAAGGGCCGGCTTTGATGGAGTTCAAATTCATGCTGTTCAT
GAAGGATACCTTATAGATCAATTTGCTATTTCATTATTTAATCATAGAACCGATGAATAC
GGCGGAAGCTTAGAAAATAGACTTCGCTTTGCAAGAGAAATCGTTGAAGAAATTAAAAA

TCGCTGTGGAGAAGATTTCCCTGTAACACTTAGATATTCACCAAAAAGCTTTATTAAAGA
TCTTAGAGATGGAGCACTTCCTGGTGAAGAATTCGTTGAAAAGGGAAGAGACCTTGAC
GAAGGTGTTGAGGCTGCAAAACTTCTTGTATCTTATGGATATGATGCTTTAGATACAGA
TGTTGGTTCTTATGATTCATGGTGGTGGAGTCATCCGCCTATGTACCAGGAAAAAGGCT
TATATAGAAAATACGCTAAATTAATGAAGGATACTGTTGATGTTCCAGTTATTTGCGCTG
GAAGAATGGATGATCCTGATATGGCCTTAGAAGCTGTAGAAAATGGAACCTGCGATGTT
ATAAGTCTAGGAAGACCTCTTCTTGCAGACCCTGACTACGTAAATAAGTTAAGAAGTAA
TAAATGCAAATCAATAAGACCTTGTATTTCCTGTCAAGAAGGTTGTATGGGACGTGTTC
AACATTACTCAATGTTAAACTGCGCTGTAAACCCTCAAGCTTGTAAGGAAAGAGCTAAC
TCACTTACTCCAATAATTAAAAGCAAAAAAGTATTAATAGTTGGAGGAGGAGTTGCTGG
CTGTGAAGCTGCTAGAGTTCTAGCTCTTAGAGGTCATGAACCTGTACTTTATGAAAAGA
GCAATAGATTAGGCGGAAATCTTATACCTGGTGGAGCACCAAGCTTTAAAGAAGATGAC
ATAGCATTAGCTGATTGGTATACAAATACCTTAAAAGAGCTAAACGTTGAAGTCAACTTA
AATAGCGAGGTTACAAAAGAACAAATTTTAAATTCCAAGTTTGATACAGTAATCGTAGCA
ACAGGATCAACTCCAAAGGTTTTCCCACTTGGAGATGACGAAAAAGTATTCACCGCTGC
TGAAGTATTACTAGGACAAAAAGATCCTGGAGAAACAACTGTTGTAGTTGGAGGAGGTC
TAGTAGGCTGCGAATTAGCATTAGATCTTGCTAAAAAAGGCAAAAAGGTAACTATTGTT
GAAGCCTTAAATAAAATACTAGCTTTAAATGGTCCTTTATGTTCTGCAAACAGCGAAATG
CTTCAAAAATTAATACCTTTTAATGGCATCGATGTAAAGGCAAATTCAAAAGTAAAAGGA
TACAAAAATGGATTGCTTAAAATGGAAACAGAAAACGGAATAGAAGAATTACCATGTGA
TTCAGTAATATTATCTGTTGGATATAAAGAAGAAAACTCCTTATACAAGGAATTAGAATT
TGAAATTCCAGAAATCTACCTTCTAGGAGATGCTCGTAAGGTATCTAATATCATGTATG
GTATTTGGGATGCTTTTGAAGTTGCAAACCATATATAA

References

1. Espah Borujeni, A. & Salis, H.M. Translation initiation is controlled by RNA folding kinetics via a ribosome drafting mechanism. *J. Am. Chem. Soc*. **138,** 7016-7023 (2016).

2. Salis, H.M., Mirsky, E.A. & Voigt, C.A. Automated design of synthetic ribosome binding sites to control protein expression. *Nat. Biotechnol*. **27,** 946 (2009).

3. Lee, T.S. *et al.* BglBrick vectors and datasheets: a synthetic biology platform for gene expression. *J. Biol. Eng.* 12 (2011).

4. Baba, T. *et al.* Construction of *Escherichia coli* K-12 in-frame, single-gene knockout mutants: the Keio collection. *Mol. Syst. Biol*. **2,** 1-11 (2006).

5. Han, L. *et al.* Engineering catechol 1,2-dioxygenase by design for improving the performance of the cis, cis-muconic acid and synthetic pathway in *Escherichia coli, Scientific Rep*. **5,** 11 (2015).

6. Niu, W., Draths, K.M. & Frost, J.W. Benzene-free synthesis of adipic acid, *Biotechnol. Progr*. **18,** 201-211 (2002).

7. Tsai, S.-C. & Li, Y.-K. Purification and characterization of a catechol 1,2-dioxygenase from a phenol degrading *Candida albicans* TL3. Arch. *Microbiol.* **187,** 199-206 (2007).

8. Vardon, D.R. *et al.* Adipic acid production from lignin. *Energy. Environ. Sci*. **8,** 617-628 (2015).

9. Matera, I. *et al.* Catechol 1,2-dioxygenase from the gram-positive *Rhodococcus opacus* 1CP: quantitative structure/activity relationship and the crystal structures of nativee and catechols adducts. *J. Struct. Biol*. **170,** 548-564 (2010).

10. Matsumura, E., Ooi, S., Murakami, S., Takenaka, S. & Aoki, K. Constitutive synthesis, purification and characterization of catechol 1,2-dioxygenase from the aniline-assimilating bacterium *Rhodococcus sp.* AN-22. *J. Biosci. Bioeng*. **98,** 71-76 (2004).
